# Exploring the Feasibility of a Telematic Version of Parkinson’s Disease—Cognitive Rating Scale (PD-CRS)

**DOI:** 10.3390/brainsci15090948

**Published:** 2025-08-30

**Authors:** Elisa Pini, Fulvio Pepe, Michelle Ingiardi, Veronica Laini, Nicoletta Ciccarelli, Eugenio Magni

**Affiliations:** 1 Fondazione Poliambulanza Istituto Ospedaliero, 25124 Brescia, Italy; elisa.pini@poliambulanza.it (E.P.); fulvio.pepe@poliambulanza.it (F.P.); michelle.ingiardi@poliambulanza.it (M.I.); veronica.laini@poliambulanza.it (V.L.); eugenio.magni@poliambulanza.it (E.M.); 2 Department of Psychology, Catholic University Milan, 20123 Milan, Italy; 3 Department of Theoretical and Applied Sciences, eCampus University, 22060 Novedrate, Italy

**Keywords:** Parkinson’s disease, cognitive assessment, cognitive screening, tele-neuropsychology, mild cognitive impairment, feasibility study, remote cognitive screening, aging, dementia prevention

## Abstract

Background/Objectives: Parkinson’s disease (PD) is a progressive neurodegenerative disorder frequently associated with cognitive impairment. In the context of increasing interest in remote healthcare solutions, particularly after the COVID-19 pandemic, this preliminary study aimed to evaluate the feasibility of an online version of the Parkinson’s Disease—Cognitive Rating Scale (PD-CRS), a first-level neuropsychological screening tool for mild cognitive impairment (MCI) in individuals with PD. Methods: Seventy-nine patients with idiopathic PD were recruited between October 2020 and February 2024. A telematic version of the PD-CRS was administered via video call using adapted materials (e.g., slide-based instructions and webcam-mediated tasks). Both patients and examiners completed a Visual Analogue Scale (VAS) to rate perceived difficulty. Descriptive statistics and non-parametric tests were used to analyze data. Results: Difficulty ratings were low for both patients (mean VAS = 1.60, SD = 0.88) and the examiner (mean VAS = 1.43, SD = 0.61), with no significant difference (*p* = 0.176). No demographic or clinical variable predicted difficulty. Conclusions: These findings support the feasibility and usability of an online version of PD-CRS. This approach may facilitate wider access to cognitive screening for PD patients, particularly those with mobility limitations or living in underserved areas.

## 1. Introduction

Parkinson’s disease (PD) is a chronic, progressive neurodegenerative disorder primarily characterized by motor symptoms such as rigidity, resting tremor, and bradykinesia. However, accumulating evidence over the past decades has underscored the complex multifaceted nature of PD, which extends far beyond motor dysfunction. Non-motor symptoms, including cognitive impairment, behavioural changes, pain, sensory deficits, autonomic dysfunction, and sleep disorders, are now recognized as integral components of the disease, often emerging even during the early stages and progressively worsening over time [1]. These non-motor manifestations frequently surpass motor symptoms in terms of their impact on the daily functioning and quality of life of patients and their caregivers [2].

Among the non-motor symptoms, cognitive impairment is particularly prevalent and clinically significant. Cognitive changes in PD may range from subtle deficits that are often underdiagnosed, to severe impairments culminating in dementia.

The intermediate stage between normal cognition and dementia is referred to as mild cognitive impairment (MCI), a condition in which overall independence in daily life is largely maintained [1]. The Movement Disorder Society (MDS) has identified MCI in PD (PD-MCI) as a frequent condition in non-demented individuals with Parkinson’s disease, associated with several factors including advanced age, longer disease duration, and increases disease severity [3]. Importantly, PD-MCI has been recognized as a major risk factor of the subsequent development of Parkinson’s disease dementia (PDD), with longitudinal studies suggesting that up to 80% of patients with PD may eventually develop dementia [4,5,6]. The diagnosis of PD-MCI requires objective evidence of cognitive decline, which can be obtained through either formal second-level neuropsychological testing or validated first-level screening tools tailored to the PD population [7]. Despite the clinical utility of comprehensive second-level assessments, their application may be limited by time, resource, and accessibility constraints in routine clinical settings.

Among the recommended first-level instruments, Litvan and colleagues (2012) [7] proposed a set of tools including the Montreal Cognitive Assessment (MoCA), the Scale for Outcomes of Parkinson’s Disease—Cognition (SCOPA-COG), the Mattis Dementia Rating Scale (MDRS) and the Parkinson’s Disease—Cognitive Rating Scale (PD-CRS) [7]. Notably, both the SCOPA-COG and the PD-CRS were specifically designed to detect cognitive dysfunctions commonly observed in PD [8].

The Italian version of PD-CRS comprises nine subtests: (1) immediate free-recall verbal memory test (score range: 0–12); (2) confrontation naming test (score range: 0–20); (3) sustained attention test (score range: 0–10); (4) working memory test (score range: 0–10); (5) unprompted drawing of clock test (score range: 0–10); (6) copy drawing of a clock test (score range: 0–10); (7) delay free-recall verbal memory test (score range: 0–12); (8) alternating verbal fluency test; (9) action verbal fluency test [9]. The test yields both a total score and domain-specific subscores. It can be administered in approximately 20–30 min and demonstrates high sensitivity for detecting executive and attentional deficits in PD.

The global outbreak of COVID-19 has brought to the forefront the necessity of remote neuropsychological assessment methods due to the widespread restrictions on face-to-face consultations. This context has accelerated interest in the field of diagnostic tele-neuropsychology, aimed at expanding access to cognitive assessment services in a more flexible, scalable, and timely manner [10]. Many patients with neurological or psychiatric conditions face logistical barriers that hinder their ability to attend in-person assessment, which may delay the diagnosis and treatment of cognitive impairments, ultimately compromising prognosis [11]. Moreover, remote assessment tools are increasingly viewed as essential for the ongoing monitoring of cognitive changes in clinical populations over time [12,13].

Recent reviews, such as that by Binoy et al. (2024), have explored the applicability of tele-neuropsychological protocols in Parkinson’s disease, demonstrating promising levels of feasibility and reliability when appropriate adaptations are implemented [14]. Among the main advantages of remote assessment are the increased accessibility for individuals with reduced mobility or those living in geographically remote areas, the continuity of care, and reduced costs for both patients and institutions. However, limitations remain, including technological barriers, reduced control over environmental distractions, and the potential loss of qualitative information derived from in-person interactions [14]. These challenges are particularly relevant in PD, where motor symptoms, hearing difficulties, and bradyphrenia may interfere with digital assessment if not properly accounted for.

Against this backdrop, the present study aims to investigate the feasibility of administering a digital, online version of the PD-CRS, with the goal of enhancing accessibility and continuity of cognitive evaluation in individuals with Parkinson’s disease.

## 2. Materials and Methods

### 2.1. Population

A total of seventy-nine patients diagnosed with idiopathic Parkinson’s disease (PD) were recruited from the fifteenth of October 2020 to the end of February 2024 during scheduled neurological follow-up visits. Written informed consent was obtained from all participants, in accordance with approval from the local ethics committee.

Inclusion criteria were as follows: diagnosis of idiopathic PD; age equal to or younger than 85 years; ability to understand and sign informed consent; absence of a formal diagnosis of cognitive impairment; access to a stable internet connection and a compatible electronic device (e.g., computer or tablet); and either sufficient digital literacy or the availability of a caregiver capable of assisting with video call setup.

At the time of neuropsychological assessment, demographic and clinical data were collected through patient interviews and medical records. The data included: age, years of education, age at disease onset, side of motor symptom onset, motor phenotype (akinetic-rigid, tremor-dominant, or postural instability and gait disorder subtype), levodopa equivalent daily dose (LED), concurrent pharmacological treatments, comorbidities, and the most recent Unified Parkinson’s Disease Rating Scale (UPDRS) scores. All patients were undergoing daily dopaminergic therapy, and they were assessed during the “on” phase of treatment.

### 2.2. Materials and Procedures

A telematic version of the PD-CRS was developed with the aim of maximizing usability and preserving the structure of the original assessment. Participants who consented to the study were first contacted by phone to arrange an appointment. Subsequently, two emails were sent: the first confirmed the date and time of the scheduled session, and the second contained the video call link. Additionally, participants received a set of preparatory instructions to ensure optimal conditions for the remote evaluation.

They were instructed to connect via computer or tablet (rather than a smartphone) to ensure optimal visibility of visual materials. Participants were also asked to have a few blank sheets of paper and a pen available during the assessment. On the day of the session, patients were permitted to involve a caregiver to assist with technical access if necessary.

A PowerPoint presentation was used to deliver the test stimuli during the video call. The examiner controlled the slide progression during the session. Before each subtest, a slide containing written instructions was presented and read aloud. In cases where the original version included examples, these were also shown in written form. All instructions were provided in both oral and visual modalities.

**Figure 1:**
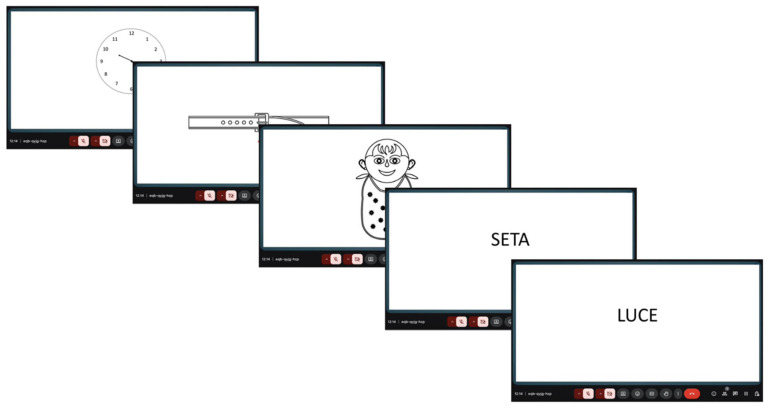
Telematic version of PD-CRS, example of same screens from the patient’s perspective.

For the Immediate Recall and Naming subtests, one stimulus was displayed per slide. During the Clock drawing subtest, participants were instructed to complete the task on paper and then display their drawing to the webcam. The examiner captured an image of the drawing to perform subsequent scoring. This adaptation allowed for the remote administration of subtests involving visual-spatial and graphic components, maintaining both usability and fidelity to the in-person format (see Figure 1).

A total of three neuropsychologists who had previously undergone specific training to ensure consistency in administration were involved in the study. To minimize examiner-related bias, the assessments were counterbalanced by adopting a consecutive rotation scheme, whereby each neuropsychologist evaluated one patient in turn (i.e., examiner A, examiner, examiner C, and then again examiner A).

**Figure 2:**
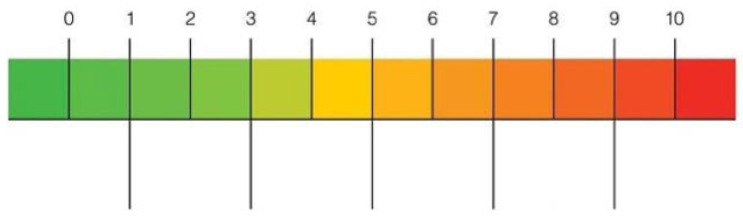
Visual Analogue Scale (VAS). Notes: 0 = “Not at all difficult”; 10 = “extremely difficult”.

At the end of the session, both the examiner and the participant completed a Visual Analogue Scale (VAS) to report perceived difficulty in completing the assessment. Ratings ranging from 0 (“not at all difficult”) to 10 (“extremely difficult”) (see Figure 2).

### 2.3. Statistical Analysis

Descriptive statistics were computed to summarize the demographic and clinical characteristics of the study sample. Specifically, qualitative variables were expressed as absolute frequencies and percentages. Quantitative variables were described as means and standard deviations. To compare Visual Analogue Scale (VAS) scores reported by patients and the examiners, the Wilcoxon Signed-Rank Test was performed. To further explore whether perceived difficulty was associated with specific demographic or clinical variables, a series of non-parametric analyses were conducted. All statistical analyses were conducted using SPSS Statistics software, version 19.0 (SPSS Inc., Chicago, IL, USA).

## 3. Results

### 3.1. Descriptive Analyses

The demographic and clinical characteristics of the sample are presented in Table 1. The cohort included seventy-nine patients with Parkinson’s disease (46 males, 33 females) with a mean age of 69 years (range: 53–83, SD = 7.38) and a mean education of 9 years (range: 5–18, SD = 3.36). The average age at disease onset was 63 years (range: 49–76, SD = 7.97). Forty-one patients (52%) exhibited right-sided motor onset, and 38 patients (48%) presented with a tremor-dominant phenotype. The mean total UPDRS score was 34.4 (SD = 13.78, range 0–59). Most participants reported minimal functional limitations (UPDRS Part II mean score = 6.48, SD = 4.40, range 0–18) and showed mild-to-moderate motor disability (UPDRS Part III mean score = 27.0, SD = 8.68, range 10–48). Although approximately 34% of the sample (*n* = 27) were on antidepressant and/or anti-anxiety medication, the mean score at UPDRS Part I (clinical evaluation of cognition, behaviour, and mood) was very low (0.99, SD = 1.2, range 0–5) suggesting overall good compensation in these domains.

### 3.2. Feasibility of the Telematic Version of the PD-CRS

**Figure 3:**
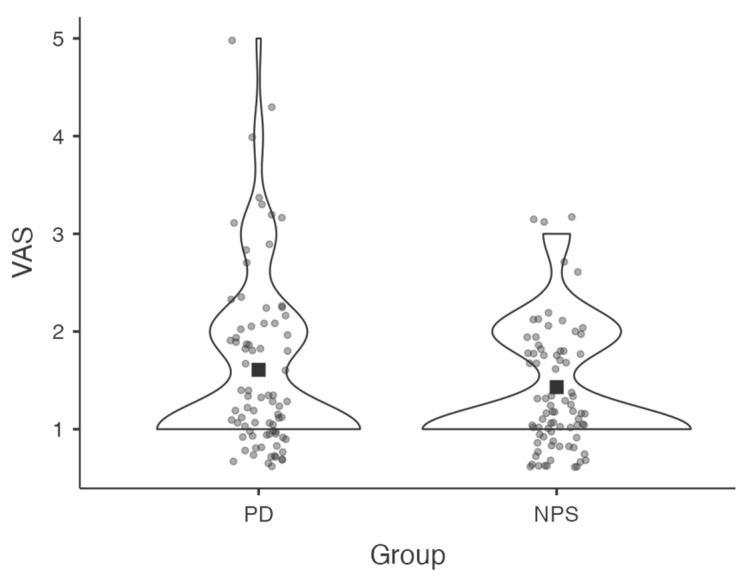
Comparison between patients and examiner on a VAS in terms of the perceived subjective difficulty in completing PD-CRS. No statistically significant difference was found between the two groups (Wilcoxon signed-rank test, *p* = 0.176). The black square represents the mean value for each group. Abbreviations: VAS Visual Analogue Scale; PD patient with Parkinson’s Disease; NPS neuropsychologist; PD-CRS Parkinson’s Disease—Cognitive Rating Scale.

To evaluate the feasibility of administering the PD-CRS remotely, both patients and examiner rated the perceived difficulty of the assessment using a Visual Analogue Scale (VAS), ranging from 0 (“not at all difficult”) to 10 (“extremely difficult”). The mean VAS score reported by patients was 1.6 (SD = 0.88), while the neuropsychologist reported a mean score of 1.43 (SD = 0.61). A Wilcoxon signed-rank test revealed no statistically significant difference between the two groups (*p* = 0.176), which indicates that no significant differences emerged in the VAS ratings provided by patients and the examiner (see Figure 3). It was also important to obtain preliminary evidence of feasibility directly from the target population, to be compared with the perception of difficulty developed by the examiner during the implementation of the tool, since patients will ultimately represent the end-users once validated. Further studies—particularly those directly comparing remote and in-person administration—are needed to confirm these findings.

To assess whether statistically significant differences in the dependent variable VAS-PD existed across groups, Mann–Whitney U tests were applied for binary variables (gender, presence of mood disorders), and Kruskal–Wallis tests for variables with more than two categories (education, side of motor symptom onset, clinical phenotype, UPDRS total score). Spearman’s rank-order correlations were used for continuous variables (age, levodopa equivalent dose, disease duration, age at diagnosis). The results, summarized in Table 2, showed that none of the variables analyzed were significantly associated with VAS scores. Age (*p* = 0.229), Education (*p* = 0.650), LED (*p* = 0.906), Disease duration (*p* = 0.486), and age at diagnosis (*p* = 0.343) were not predictive of perceived difficulty. Similarly, no significant associations were found between VAS scores and UPDRS total score (*p* = 0.796). Furthermore, no differences in VAS scores were observed between males and females (*p* = 0.336), or between patients with and without mood disorders (*p* = 0.600), nor among groups differing in clinical phenotype (*p* = 0.485) or side of motor onset (*p* = 0.901). Taken together, these findings may suggest that the telematic version of the PD-CRS was perceived with minimal reported difficulty across the sample. The absence of significant association between VAS scores and clinical severity, motor symptoms, or demographic factors indicates that perceived feasibility was broadly comparable among the investigated subgroups. Further studies, particularly those including direct comparisons with in-person administration, are needed to confirm the overall feasibility and applicability of this approach in both clinical and remote assessment contexts.

## 4. Discussion

This study aimed to assess the feasibility of a telematic version of the Parkinson’s Disease—Cognitive Rating Scale (PD-CRS) as a first-level screening tool for detecting mild cognitive impairment (MCI) in individuals with idiopathic Parkinson’s disease (PD) without overt dementia. Following remote administration, both patients and the examiner rated the perceived difficulty of completing the assessment using a Visual Analogue Scale (VAS).

The primary finding of this preliminary study is that the telematic version of PD-CRS was well tolerated and perceived as minimally difficult by both patients and the examiner. There results underscore the potential of digital tools to support cognitive screening and monitoring in older populations, even among individuals with limited technological familiarity. The low VAS scores from both groups highlight the acceptability and usability of the online version, particularly in the contexts where face-to-face assessment are not feasible, such as during the COVID-19 pandemic [10,11,12].

Crucially, the success of the remote protocol was facilitated by several structural supports, including clear written and oral instructions, the option to involve a caregiver for technical assistance, and the use of standardized visual materials (e.g., PowerPoint slides). Tasks requiring visuospatial or motor output—such as the Clock Drawing Test—were successfully adapted by having participants draw on paper and display their output via webcam, allowing for real-time scoring without compromising the integrity of the test.

One possible objection is that the low difficulty score reported by the examiner on the VAS may partly reflect a learning effect in administering the tool. However, the equally low VAS scores obtained from patients, who were all undergoing the procedure for the first time, support a different interpretation, namely that the instrument itself is feasible for use in this population. Moreover, it should be emphasized that perceived difficulty on the VAS also encompasses the technical and logistical aspects inherent to the telematic administration of a scale (e.g., unstable internet connection, device-related problems). Paradoxically, the more often the instrument is administered, the higher the likelihood of encountering such technical issues, which could in turn increase rather than decrease the examiner’s perceived difficulty over time.

Importantly, results from the secondary analyses (see Table 2) demonstrated that no demographic or clinical variable (including age, education, disease severity, mood disorders or motor phenotype) was significantly associated with perceived difficulty during the assessment. This suggest that the telematic PD-CRS is equally feasible across a wide range of patient profiles, including those with more advance motor symptoms or lower education levels. Such robustness reinforces the potential of this format to be broadly implemented in clinical settings, regardless of individual variability in disease expression or demographic background.

From a clinical perspective, the early identification of cognitive impairment in PD remains a priority, as PD-MCI is a common condition that often precedes dementia.

The Movement Disorder Society Task Force estimates that approximately 26.7% of individuals with PD develop MCI, with a considerable proportion progressing to dementia in the long term [3,5,7]. In this context, having access to brief, sensitive and scalable tools like the PD-CRS is essential for timely detection and intervention [8,9].

Our findings are consistent with the growing literature supporting the feasibility and acceptability of remote neuropsychological evaluations, particularly when combined with caregiver support and digital aids [13]. Remote tools may play a vital role in promoting healthcare equity by improving access for individuals with physical disabilities or those living in geographically isolated areas.

Nonetheless, several limitations should be acknowledged. First, the study’s feasibility assessment is limited to individuals with access to digital devices and internet connectivity, potentially excluding more vulnerable subgroups of the PD population. Second, the cross-sectional and observational design of the study precludes any conclusions regarding cognitive trajectories or predictive validity. Future studies with a longitudinal design are needed to evaluate whether the online PD-CRS can sensitively monitor cognitive changes over time and predict development of Parkinson’s disease dementia (PDD). Additionally, research should directly compare the telematic and in-person version of the PD-CRS to assess their equivalence in terms of psychometric properties and diagnostic accuracy.

Finally, it is important to emphasize that the present study represents a preliminary phase of a larger research project aimed at validating the telematic version of the PD-CRS. Building on these encouraging feasibility results, future investigations will focus on confirming the reliability, construct validity, and diagnostic performance on the online tool. Particular attention will also be given to refining administration procedures and expanding its applicability to broader and more diverse clinical populations, including that whit limited digital literacy or reduced access to technology.

## 5. Conclusions

Following the COVID-19 pandemic, there has been increasing interest in the application of tele-neuropsychology for diagnostic and monitoring purposes. This study provides preliminary evidence supporting the feasibility of implementing an online version of the Parkinson’s Disease—Cognitive Rating Scale (PD-CRS) as a first-level screening tool for the detection of mild cognitive impairment (MCI) in individuals with Parkinson’s disease. The online administration was well tolerated and easily accessible, even among older adults with limited digital experience. This telematic approach has the potential to improve access to cognitive assessments, particularly for patients with significant motor disabilities or those residing far from specialized clinical centres, thereby promoting more inclusive and continuous cognitive monitoring in clinical practice.

Moreover, both patients and the examiner reported minimal difficulty in completing the assessment, and no significant associations were found between perceived difficulty and demographic or clinical variables. These findings suggest that the telematic PD-CRS may be a promising and user-friendly tool for remote neuropsychological evaluation, particularly in contexts where in-person assessments are not practical or possible.

While these initial results are encouraging, they should be interpreted considering the study’s limitations. As a feasibility study, this work constitutes the first step in a broader validation process. Future research is warranted to examine the psychometric properties, diagnostic accuracy, and longitudinal applicability of the telematic PD-CRS. If validated, this tool could enhance early detection of cognitive impairment in PD and promote more equitable access to neuropsychological services across diverse populations and healthcare settings.

## Figures and Tables

**Table 1:** Patients’ characteristics.

Variables	N (%) or Mean (SD)
Age (years)	69.3 (7.38)
Education (years)	9.3 (3.36)
Age at time of diagnosis (years)	63.0 (7.97)
Right side motor onset	41 (51.9%)
Tremor dominant subtype	38 (48.1%)
Akinetic-rigid subtype	36 (45.6%)
UPDRS I (range 0–16)	0.9 (1.2)
UPDRS II (range 0–52)	6.8 (4.40)
UPDRS III (range 0–56)	27.0 (8.68)
UPDRS IV (range 0–23)	0.99 (1.69)
UPDRS total (0–147)	34.4 (13.78)
LED (mg)	622.4 (474.51)
Antidepressant and/or anti-anxiety medication	27 (34.2%)

Abbreviations: N Number, SD Standard Deviation; LED levodopa equivalent dose. Notes: Continuous variables are presented as mean (SD); categorial variables are presented as number (%).

**Table 2:** Association between demographic and clinical variables and perceived difficulty during telematic administration of the PD-CRS.

Independent Variable	Statistic	Test	gdl	*p*-Value
Age	Spearman’s ρ	−0.137	77	0.229
Education	Kruskal–Wallis H	8.63	11	0.650
Gender	Mann–Whitney U	673	-	0.336
Led (mg)	Spearman’s ρ	0.014	77	0.906
Side of onset	Kruskal–Wallis H	0.209	2	0.901
Clinical phenotype	Kruskal–Wallis H	2.45	3	0.485
Mood disorders	Mann–Whitney U	679	-	0.600
UPDRS total	Kruskal–Wallis H	29.7	37	0.796
Disease duration	Spearman’s ρ	−0.080	76	0.486
Age at time of diagnosis	Spearman’s ρ	−0.109	76	0.343

Abbreviations: Led Levodopa equivalent dose; UPDRS Unified Parkinson’s Disease Rating Scale; Spearman’s p Spearman’s rank-order correlation matrix; Mann–Whitney U Non-parametric independent samples comparison (Mann–Whitney U test); Kruskal–Wallis H Kruskal–Wallis one-way analysis of variance.

## Data Availability

The data that support the findings of this study are available from the corresponding author upon reasonable request due to restrictions for privacy.
